# Late-Onset OCD as a Potential Harbinger of Dementia With Lewy Bodies: A Report of Two Cases

**DOI:** 10.3389/fpsyt.2020.00554

**Published:** 2020-06-30

**Authors:** Solène Frileux, Bruno Millet, Philippe Fossati

**Affiliations:** ^1^ Control-Interoception-Attention Team, Brain Institute of Paris, Paris, France; ^2^ Faculty of Medicine, University of Sorbonne Universités UPMC, Paris, France; ^3^ Department of Psychiatry, University Hospital La Pitié Salpêtrière, Paris, France

**Keywords:** late-onset, obsessive-compulsive disorder, dementia with Lewy bodies, behavioral psychological symptoms of dementia, neuropsychiatric disorders

## Abstract

**Objectives:**

Obsessive-compulsive disorder usually begins in adolescence or young adulthood. OCD cases appearing after the age of 50 years are rare, most often associated with inflammatory, brain lesions, or neurodegenerative comorbidities. We describe two cases of late-onset obsessive compulsive disorder followed by the development of Dementia with Lewy Bodies and review the links between these two disorders.

**Methods and Results:**

We describe the clinical history of two patients that first showed OCD symptoms at an atypical age (>60 years). After several failed treatment attempts, they were hospitalized in our unit. Both presented severe sensitivity to antipsychotic agents that led to a diagnosis of Dementia with Lewy Bodies. Administration of cholinesterase inhibitors was associated with decrease of psychiatric symptoms in both cases. In addition to those clinical observations, a systematic review of the literature suggests that, beyond prefrontal cortex, temporal lobe and putamen have important roles in OCD pathophysiology. Based on these findings, we discuss four hypotheses to explain the sequential appearance of OCD and DLB symptoms. First, we considered the possibility that comorbidity of OCD with DLB was coincidental. Second, we propose to interpret OCD symptoms as motor stereotypies. Third, we hypothesize that late-onset OCD might be a symptom of late-onset depression. Four, we hypothesize that through early deterioration of basal ganglia, DLB caused the onset of OCD.

**Conclusion:**

In conclusion, we recommend that cases of late-onset treatment-resistant OCD should be carefully tested for possible organic etiologies, and for DLB in particular.

## Introduction

Almost all cases of obsessive compulsive disorder (OCD) begin before the age of 35 years (1, 2). Out of a cohort of 1000 OCD patients, Weiss et al. only reported 5 cases beginning after 50 years (3). Some authors have highlighted that early- and late-onset OCD show different profiles in terms of familial history, clinical picture, and comorbidity patterns (3, 4). Even though there is no clear consensus on the cutoff age to define early and late-onset OCD, age of onset might clearly influence clinical phenotype, and associated features of OCD.

For instance, late-onset OCD is often associated with neurological diseases—such as frontotemporal dementia, progressive supranuclear palsy, Huntington's disease, or focal cerebral lesions (5–8).

Dementia with Lewy Bodies is identified as the second most common type of dementia (9) and represents a major health challenge (9). Its diagnosis and accurate treatment are often delayed as many patients are first diagnosed with a psychiatric disorder. A recent review by McKeith et al. emphasized the current lack of information regarding psychiatric-onset types of DLB, while such information would help to diagnose DLB at a pre-dementia stage (10). In particular, there are few reports with detailed description of clinical courses observed in psychiatric practice (10). Here we present two clinical cases of late-onset OCD followed by the development of Dementia with Lewy Bodies (DLB). Symptomatic treatment of DLB resulted in OCD improvement. We then review the literature discussing the putative links between these two disorders in order to shed light on the pathophysiology of late-onset OCD in particular and OCD in general.

## Case Reports

### First Case: Patient #1

A 60-year-old man, university professor with no past medical history, developed OCD symptoms. His father had suffered from hypochondriac delusions and one of his cousins had committed suicide. This patient had first excessive preoccupations of contamination and infection at age of 60 years. Behavioral alterations included cleaning compulsions, dishwashing rituals, and boiling forks and knives before every meal. He retired earlier than expected due to the impact of these symptoms. He consulted a psychiatrist at age 62 years and was treated for 9 years with three consecutive SSRI drugs, without improvement.

In August 2016, at age 74 and 14 years after onset of OCD symptoms, the patient was admitted to the psychiatry department of Pitié-Salpêtrière Hospital. At this point, he suffered from severe depression with delusional symptoms focused on his intestinal transit. Further, since the age of 71 years he showed an extrapyramidal syndrome characterized by bradykinesia, dysphonia, and occasional falls due to mild ataxia. During his stay at the hospital cognitive impairments were observed as well, namely memory and attention difficulties.

The patient was initially prescribed 150 mg of clomipramine. 5 days later, 7.5 mg of olanzapine was given added to the prior pharmacological treatment. However, shortly after olanzapine introduction, he deteriorated with aggravation of the extrapyramidal syndrome, severe confusion, visual hallucinations (children passing by very quickly), sleep disorders, orthostatic hypotension, swallowing difficulties, and dyspraxia.

**Figure 1:**
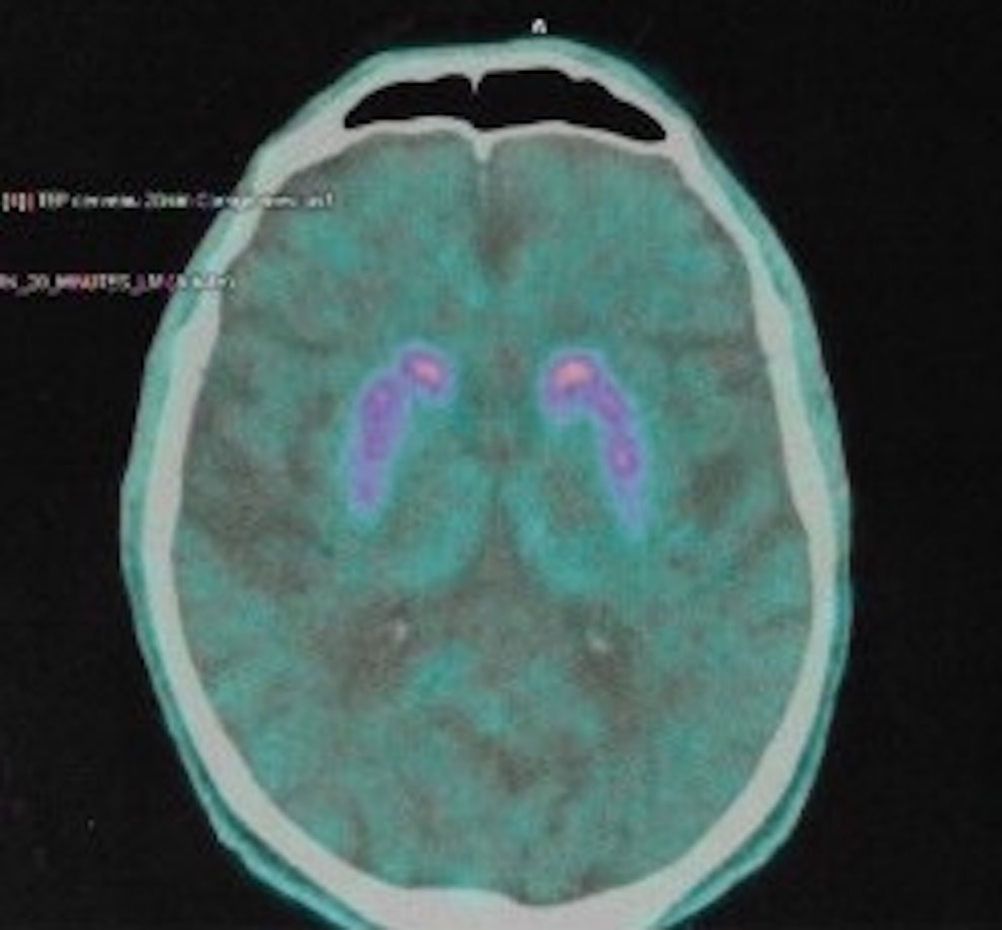
DaT-SCAN SPECT from patient #1, showing reduced striatal uptake, asymmetric, predominant in putamen.

Magnetic resonance imaging (MRI) of the brain showed predominantly cortical atrophy (see **Supplementary Materials** for details). Single-photon emission computed tomography (SPECT) neuroimaging showed bilateral hypometabolism in associative and visual cortical regions. DaT (dopamine transporter)—scan imaging revealed a symmetric presynaptic putamen dopaminergic denervation (**Figure 1**).

Interruption of antipsychotic treatment led to a progressive improvement in temporo-spatial orientation, confirming neuroleptics hypersensitivity. Clomipramine was interrupted due to orthostatic hypotension and dysuria. Once psychiatric symptoms were improved, neuropsychological examination was run and showed a moderate cognitive deterioration: the patient had an impairment of verbal anterograde episodic memory and increased sensitivity to attentional interferences. Consolidation of long-term information was preserved, suggesting the absence of hippocampal amnesia. He also showed a severe deficit of sustained attention, a dysexecutive syndrome, and signs of apraxia. His Mini-Mental State Examination (MMSE) score was 24/30 (see **Supplementary Materials** for details). These signs hinted at a cortico-subcortical disease.

The diagnosis of probable DLB was confirmed according to the revised criteria for the clinical diagnosis of DLB. Dementia criteria were met. First, patient #1 probably retired earlier than expected because of impaired job performance. Over the last months, he showed severe attentional disorders interfering with daily functions (forgot to turn off the taps after use, left the front door open) and concentration problems that prevented him from reading complex books as before. Measures of executive function and attention were impaired. Also, patient #1 presented two core clinical features (namely fluctuating cognition and parkinsonism), five supportive clinical features (namely sensitivity to antipsychotic agents, postural instability, repeated falls, autonomic dysfunction, and depression), one indicative biomarker (reduced dopamine transporter uptake in basal ganglia by SPECT), and two supportive biomarkers (relative preservation of medial temporal lobe structures on MRI scan, generalized low uptake on PET metabolism scan with reduced occipital activity) (9). Others DLB prodromal symptoms include dysautonomia, olfactory dysfunction, rapid eye movement sleep behavior disorder (RBD) (11). The patient suffered from resistant constipation and repeated falls when he was admitted, but no other DLB usual prodromes were found in his history.

After confirming the diagnosis, rivastigmine (1.5 mg bid) was introduced. Over the following two months, OCD symptoms abated: obsessions almost disappeared, with only few rituals remaining. Gait disorders improved, bradykinesia decreased, and hallucinations did not recur. However, high distractibility persisted and patient needed assistance with dressing and toileting. Depressive symptoms remained (feelings of uselessness and reduced self-esteem), thus justifying the introduction of venlafaxine, gradually increased up to 112.5 mg/day. The patient was discharged in November 2016 and remained under venlafaxine, rivastigmine, and benzodiazepines.

Over the following months, patient #1 clinically further improved: obsessions and attention deficits decreased, while motivation and euthymia reappeared. A neuropsychological assessment six months after discharge showed a global improvement, except for learning capacities and problem solving (see **Supplementary Materials** for more details). While such improvement in neuropsychological performance is rather unusual in DLB, one could hypothesize that initial poor performance was partly due to cognitive side effects of psychotropic drugs, especially clomipramine with its anticholinergic properties. Also, the introduction of cholinesterase inhibitor during hospital stay surely contributed to improve cognitive functions.

A treatment with venlafaxine (300 mg/day) led to further decrease of the remaining depression symptoms and the patient resumed euthymia with only few OCD signs remaining. Six months after discharge, the patient had regained interest in certain former hobbies (listening to the radio, going out). He was now able to shake hands, hug his entourage, and critically reflect on his obsessions with cleanliness. This improvement with regard to depression and OCD was tempered by DLB symptoms and persistent apathy. Indeed, patient #1 had not returned to pre-dementia cognitive level. He suffered severe attentional fluctuations. While he used to write books and to teach as a university professor, he was not able to work again. He avoided visits with friends because of inability to carry on a conversation with former colleagues. He showed difficulties in reading time on a clock and still needed daily home help for eating meals and bathing.

In July 2017, 8 months after discharge, obsessions and associated rituals were present less than one hour per day. His Y-BOCS score was 10 (4 + 6). The patient said that he was less worried about dirt, but difficulties in dressing persisted due to contamination obsessions. On the other hand, hallucinations reappeared and increased progressively from 2018. Eighteen months after discharge, he complained of growing anxiety and fluctuating mood. This led to the prescription of mirtazapine 15 mg/day. He also reported that he was not sure whether he was in the real world or was abused by an hallucination. Two years after discharge, the patient was hospitalized with acute pneumonia from which he deceased.

### Second Case: Patient #2

A 65-year-old man, executive director, with a medical history of high blood pressure, right cardiac ventricle insufficiency, and coronary bypass, came for the first time to our psychiatry unit with dysphagia obsessions and a constant feeling that his saliva was “too thick and impossible to swallow”. These symptoms appeared shortly at age of 60 years after his family was financially ruined when his firm underwent fraudulent bankruptcy. Thirty years ago, the patient consulted a psychiatrist for anxiety—fearing that he might lose one of his children. There was no other history of psychiatric illness.

A few weeks after the bankruptcy and the early obsessive thoughts followed several other symptoms:

- compulsive and day-long spitting of saliva with decrease of nutritional intake, leading to a 50-kg weight loss within 3 years,
- suicidal thoughts,
- inability to socialize, self-neglect, home confinement.

He consulted several psychiatrists, with no improvement in symptoms, followed EMDR sessions, consulted a hypnotherapist, and took several SSRI drugs with poor treatment adherence and no benefit. Gastrointestinal endoscopy was normal. He was first admitted to psychiatric care at age 65 years with a diagnosis of depressive disorder with delusional symptoms, and he was prescribed paroxetine 50 mg/day. During this one-month stay, the patient's obsession with saliva was considered a delusional symptom. Introduction of paroxetine led to a transient improvement of depression, with no effects on compulsions. Depressive symptoms resumed and additional phobias appeared: fears of water and razors, fear of suffocation.

He returned to our psychiatry unit at age 69 years. Upon admission, he demonstrated anhedonia, negligence, attentional disorder, sleep problems, and morbid thoughts. He suffered from insomnia and fell asleep while seated in a chair, because he feared suffocating while in bed. He fractioned his oral intake into five meals a day. His subjects of conversation were focused on his saliva consistency and swallowing. The patient was given first sertraline 200 mg/day and later olanzapine 10 mg/day, followed by the development of these symptoms:

- extrapyramidal syndrome and reduced facial expression, dyskinesia, gait disorder, repeated falls,
- irritability, suicidal thoughts,
- pareidolia (visual hallucinations of human heads)

Medications were reduced to sertraline 100 mg and olanzapine 7.5 mg. The diagnosis of DLB was evoked. Blood samples and EKG were normal. Neuropsychological assessment at that time showed:

- preservation of overall cognitive efficiency (MMSE 28/30),
- efficiency of episodic verbal memory, with fragile consolidation,
- preservation of temporo-spatial orientation
- dysexecutive syndrome at the instrumental level
- alteration of visuospatial and visuo-perceptive abilities (see **Supplementary Materials** for precise scores).

**Figure 2:**
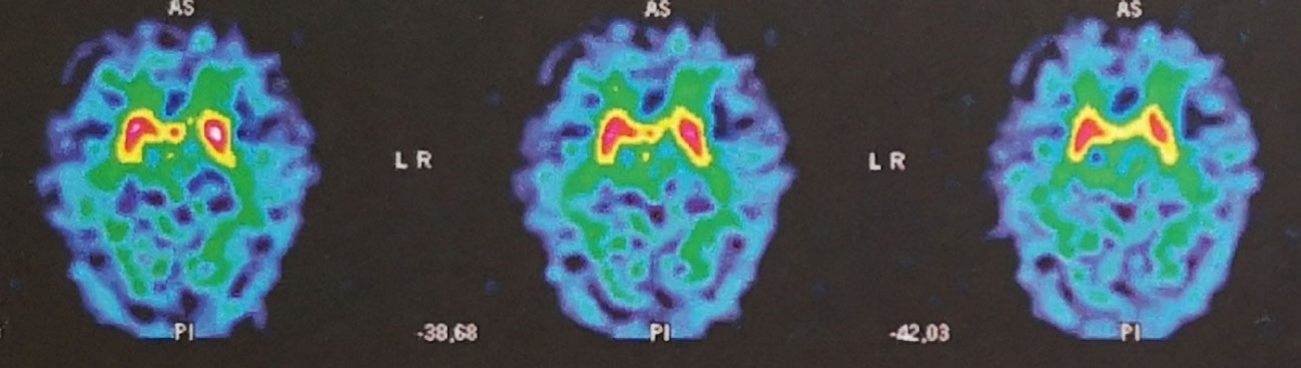
DaT-SCAN SPECT from patient #2, showing bilateral reduced striatal uptake, predominant in putamen, in favor of presynaptic dopaminergic denervation.

A brain MRI scan showed cortico-subcortical atrophy and microbleeds. A DaT-scan (**Figure 2**) showed a bilateral presynaptic dopaminergic denervation. SPECT showed a bilateral striatal fixation decrease, centered on the putamen. According to the revised criteria for the clinical diagnosis of DLB, a diagnosis of probable DLB was retained. Dementia criteria were met. Patient #2 was no longer able to do normal social and occupational functions. He did not see his friends anymore. As early as 2014, impaired figure copying (overlapping pentagons) was observed, as well as troubles in clock drawing. Neuropsychological testing run in our unit showed deficiencies in tests of visuospatial and visuoperceptual ability. Also, patient #2 presented 1 core clinical feature (recurrent visual hallucinations), two supportive clinical features (severe sensitivity to antipsychotic agents and depression), one indicative biomarker (reduced dopamine transporter uptake in basal ganglia by SPECT) and one supportive biomarker (relative preservation of medial temporal lobe structures on MRI scan) (9). Rapid eye movement sleep behavior disorder (RBD) often precedes the clinical diagnosis of DLB as well (11). Here, RBD was suspected, since the patient's wife reported restless nights, during which the patient was agitated. However, we do not have any confirmatory tests for RBD in the medical records.

Cholinergic treatment was initiated with 9.5 mg of rivastigmine and gradual introduction of levodopa.

The mood status of the patient greatly improved. Sleep was normalized, even if early awakenings sometimes persisted. Eating behavior improved. Appetite remained modest but was improved compared to the beginning of his hospitalization. Compulsive spitting persisted, but became less frequent, especially when the patient's attention was focused elsewhere. He was discharged after 6 months of stay with levodopa 62.5 mg bid, rivastigmine 9.5 mg, sertraline 100 mg, and anxiolytics.

Six months after discharge, he was brought to the emergency room by neighbors because of asthenia, self-neglect, and lack of home maintenance. Indeed, once alone at home, he was not carrying out the activities of daily life. He did not shop for food, could not complete everyday tasks, such as bathing, dressing appropriately, and preparing simple meals. Examination on admission revealed that he had stopped taking his medications, was time-disoriented, and had visual hallucinations. After a short stay, he agreed to resume his drug treatment. Home help was debuted to ensure that he was taking his medication on a daily basis, and to provide assistance with bathing, as he tended to neglect self-hygiene otherwise. Patient #2 remained stable and well as long as he was under the care of home nurses. Some feeling of dysphagia persisted. The last follow-up was at age 72 years: the patient remains concerned about saliva but does not have expectorating rituals anymore. DLB symptoms remain stable.

## Discussion

We have described two cases of treatment-resistant and late-onset OCD (i.e., onset after 50 years), followed by the development of DLB. The specific treatment of DLB allowed a partial improvement of obsessive-compulsive symptoms. Based on the observation of these cases, we suggest a possible link between these two disorders, through the following hypotheses. However, we have only observed a small number of cases, and this question needs to be further assessed in patients with late-onset OCD.

### Comorbidity of OCD With DLB Might Be Incidental

The possibility that comorbidity of OCD with DLB was just coincidental should be considered. OCD existed for many years before DLB symptoms onset in both patients. They both had psychiatric family history or clinical history. In a large case series, Weiss et al. noticed that late-onset OCD often occurred in combination with brain lesions (3). Other reports have described cases in which dementia appeared several years after a diagnosis of late-onset OCD (12, 13). Neural basis might originally have an affinity for occurrence of psychiatric symptoms.

Treating patients suffering late-onset psychiatric symptoms by cholinesterase inhibitors could still be discussed, even if they do not fulfill the criteria for DLB. Indeed, a decrease of cholinergic interneurons has been observed in OCD patients' striatum (14). In a recent study, suppression of cholinergic interneurons in rats striatum led to the development of repeated ritualized behavior (15). Several reports have pointed out the possible role of cholinergic disturbance in neuropsychiatric manifestations of dementia (16, 17). It appears that acetylcholinesterase inhibitors have psychotropic effects and might play a beneficial role in controlling behavioral and psychological symptoms of dementia (BPSD) such as agitation, apathy, and psychosis (18). Here we suggest exploring these effects to OCD symptoms in future studies.

### Were Obsessive-Compulsive Symptoms Caused by DLB-Related Frontal Stereotypies?

The main difference between stereotypies and OCD lies in the function of the symptom: relief of distress caused by the obsessive fears for OCD, automaticity without goal for stereotypies.

DLB is associated with fronto-striatal lesions. Frontal lobe dysfunction has been associated with BPSD, including motor stereotypies (cluster “aberrant motor activity” in the Neuro Psychiatric Inventory questionnaire) (19). While their prevalence has never been specifically studied in DLB, they show the highest frequency/severity score in Alzheimer's disease (19). One could imagine that fronto-striatal lesions in DLB generate BPSD, such as repetitive impulsions. However, Moheb et al. made a distinction between stereotypies and OCD in their study on frontotemporal dementia: patients suffering from dementia showed a low frequency of cleaning and ordering behaviors, and repetitive impulsions were not associated with verbalized anxiety or obsessional ideation (20). In contrast, it was possible to discuss the obsessions underlying their compulsions with our patients, and they explained their compulsive behavior by trying to reduce their anxiety. Further, in fronto-temporal dementia, repetitive behaviors are often associated with disinhibition—especially in social domain (20), which we never observed in our patients.

Impulse Control Disorder could also be evoked as a differential diagnosis. This syndrome shares clinical features with OCD, namely « difficulties resisting the urge to engage in specific behaviors that interfere with functioning » (21). The underlying question is the distinction between impulsivity and compulsivity. Impulsive behaviors are often comorbid with cluster B personality disorders (22). Further, while compulsivity leads to accomplish an unpleasant ritual in an attempt to alleviate anxiety, impulsivity is generally driven by the desire to obtain pleasure (23). None of our patients had a B-type personality, although we did not use structured clinical interview to assess personality traits or disorders. In both cases, the rituals did not provide any pleasure but relieved negative affect.

### Could the Observed Obsessive-Compulsive Symptoms Be Due to Late-Life Depression?

Depression is common in later life, affecting nearly 13% in adults 80 years and older (24–26). In DLB, up to 40% of patients show at least one depressive episode over the course of their illness (27). In our two cases, clinical diagnosis of depressive disorder with delusion could be considered, given the high frequency of somatic delusion in elderly depressed patients.

Late-onset depression often occurs in a context of disability, cognitive dysfunction, and psychosocial adversity (25). Prior to appearance of OCD symptoms, patient #1 had difficulties in his professional activities, and patient #2 faced a dismal professional situation impacting his family. In addition, the depressive symptoms observed in the two patients could be explained as a reaction to a perceived cognitive decline (26).

Depression among elderly patients is also known to feature different symptoms than in middle-aged patients, typically including more somatic symptoms (26, 28). From this viewpoint, the herein described obsessions about gastrointestinal transit and saliva, with consecutive compulsions, could be the consequence of severe depressive episodes among our patients.

However, we think that this interpretation is unlikely, since OCD is rarely observed in late-life depression (29). In a series of 336 elderly MDD patients, only 0.3% showed obsessive-compulsive symptoms (30). In addition, both patients had obsessions and compulsions long before the first depressive symptoms appeared.

### Is Late-Onset OCD an Early Sign of DLB?

McKeith et al. recently emphasized the importance to diagnose DLB at the prodromal stage, when core features might still be absent and when biomarker evidence may be weaker (10). McKeith et al. aimed to characterize three kinds of prototypic prodromal DLB syndromes, including the “psychiatric disorder DLB”: with primary presentation as late-onset affective disorder or psychosis, typically treatment refractory and showing high intolerance to neuroleptics (10). While late-onset major depressive disorder and late-onset psychosis are the most frequently reported presentations in these cases, OCD were not mentioned in this previous review.

However, several parallels exist between DLB and OCD. In both disorders, similar patterns of brain lesions can be found. DLB is associated with cortical atrophy involving frontal, temporal, parietal lobes (31), and the putamen (32, 33); nigrostriatal degeneration and subsequent dopamine transporter loss can be observed in putamen and caudate nucleus (34, 35).

Frontal regions and cortico-striatal-thalamic pathways are involved in the pathophysiology of OCD (36–38). Examples of putamen lesions followed by development of severe OCD suggest a role for this structure in OCD as well. Dopaminergic dysfunction is also part of OCD, with decreased D2- and D3- receptors availability observed in putamen (39). In our patients the dopaminergic dysfunction integral to DLB may have facilitated the emergence of OCD symptoms.

### Patients' Perspective

Patient #1 was always able to criticize his symptoms, but was initially too depressed to be aware of the links between his two disorders. He was able to regain some autonomy once OCD decreased, which helped improve his mood. He was able to express satisfaction in exerting some of his previous activities again. Cognitive impairment was particularly difficult for him to accept, given his previous high intellectual level.

Patient #2 always demonstrated weak insight, despite OCD improvement. He was able to notice that his daily life improved, but without making a connection with the dementia treatment. Further, he remained poorly compliant with treatment in the absence of nurses.

## Conclusion

We present two rare clinical pictures that raise questions about the physiopathological and anatomical bases of late-onset obsessive-compulsive disorder. We discuss several potential explanations of the sequential appearance of OCD and DLB symptoms in our patients.

First, we considered the possibility that comorbidity of OCD with DLB was coincidental. Second, we proposed to interpret OCD symptoms as motor stereotypies, which would be the consequences of the frontal damage inherent to DLB. Third, we hypothesized that late-onset OCD in our patients may be a symptom of late-onset depression. Four, we hypothesized that through early deterioration of basal ganglia, DLB leads to putamen dysfunction, causing the onset of OCD long before cardinal symptoms of dementia. We favor this last explanation.

We conclude that OCD that is treatment resistant and has started after the age of 50 years, should systematically lead the clinician to consider dementia, and more specifically to test for a potential Dementia with Lewy Bodies.

## Data Availability Statement

The original contributions presented in the study are included in the article/**Supplementary Material**; further inquiries can be directed to the corresponding author.

## Ethics Statement

Written informed consent was obtained from the individual(s) for the publication of any potentially identifiable images or data included in this article.

## Author Contributions

SF: Formal analysis; Investigation; Roles/Writing—original draft; Writing—review and editing. BM: Conceptualization; Project administration; Visualization. PF: Conceptualization; Formal analysis; Methodology; Project administration; Supervision; Validation; Visualization; Roles/Writing—original draft; Writing—review and editing.

## Conflict of Interest

The authors declare that the research was conducted in the absence of any commercial or financial relationships that could be construed as a potential conflict of interest.

## Supplementary Material

Image 1 Patient #1 Timeline : historical and current information from this episode of care.

Image 2 Patient #2 Timeline: historical and current information from this episode of care.

Image 3 Complementary sequences of DaT-SCAN SPECT from Patient #1, showing decreased dopamine concentrations in both putamen structures.

Image 4 Complementary sequences of DaT-SCAN SPECT from Patient #1, showing decreased dopamine concentrations in both putamen structures.

Image 5 Brain MRI from Patient #2 in 2014. Cerebral microbleed can be observed in left internal capsule.

Image 6 Brain MRI from Patient #2 in 2016, at time of admission, showing cortical and sub-cortical atrophy. Superficial and deep cerebral microbleeds are observed.

Image 7 Complementary sequences of DaT-SCAN SPECT from Patient #2, showing significant bilateral reduction in putamen uptake

Table 1 Patient#2 Neuropsychological Assessment in 2016. This assessment has been realized two months after patient's admission in psychiatry unit, once neuropsychiatric symptoms were stabilized (under rivastigmine 9 mg, olanzapine 5 mg and sertraline 100 mg daily).

## Abbreviations

BPSD, behavioral and psychological symptoms of dementia; DaT (dopamine transporter) – Scan; FTD, fronto-temporal dementia; DLB, Dementia with Lewy Bodies; MMSE, Mini-Mental State Examination; OCD, obsessive-compulsive disorder; RBD, Rapid eye movement sleep Behavior Disorder; SPECT, Single-photon emission computed tomography (SPECT).
