# Supplementary figures and images for: Late-Onset OCD as a Potential Harbinger of Dementia With Lewy Bodies: A Report of Two Cases
Source: Front Psychiatry. 2020 Jun 30;11:554. doi: 10.3389/fpsyt.2020.00554 (PMC7344181; doi:10.3389/fpsyt.2020.00554)

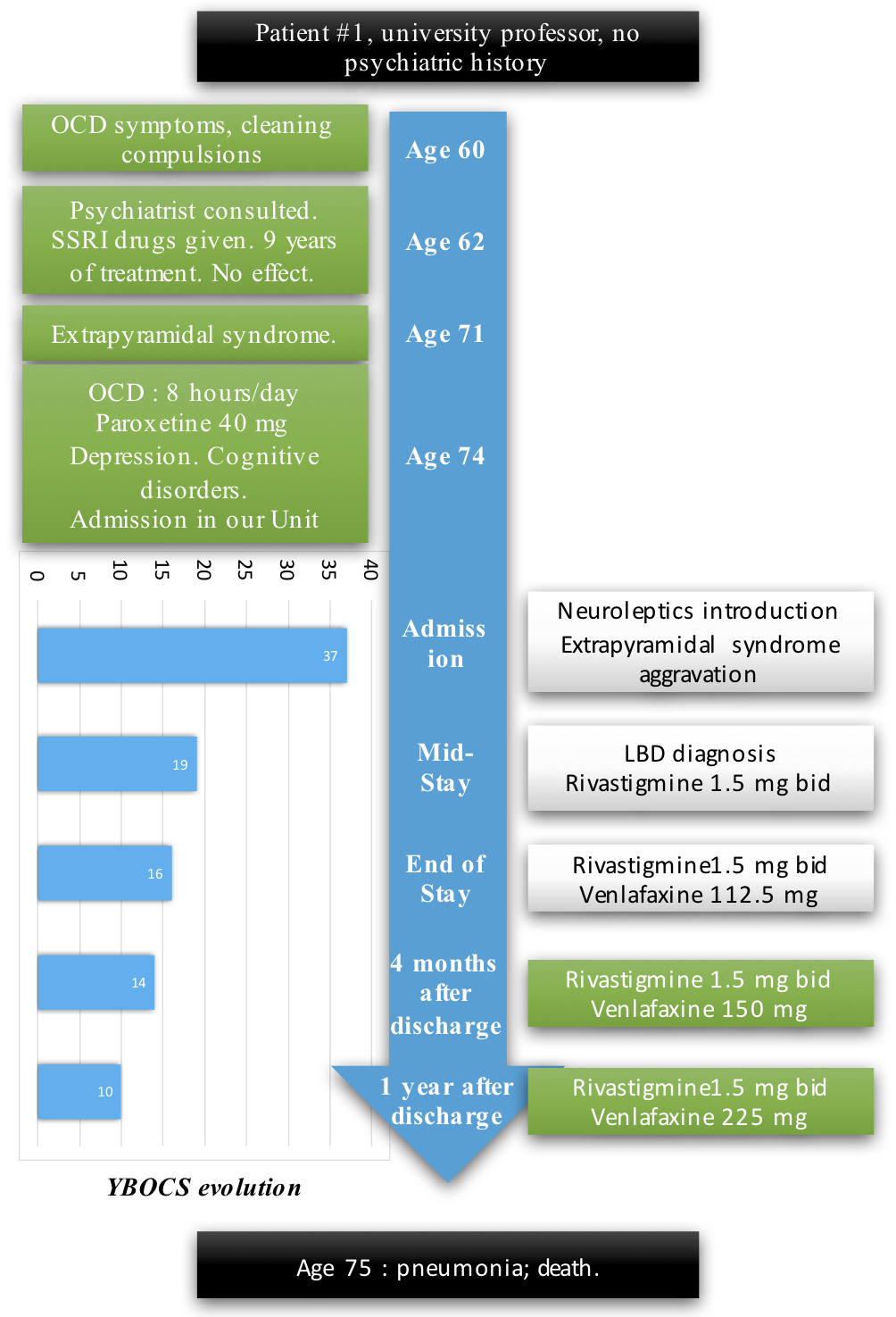

Supplement: Image 1 — Patient #1 Timeline : historical and current information from this episode of care. [file Image_1.jpeg]

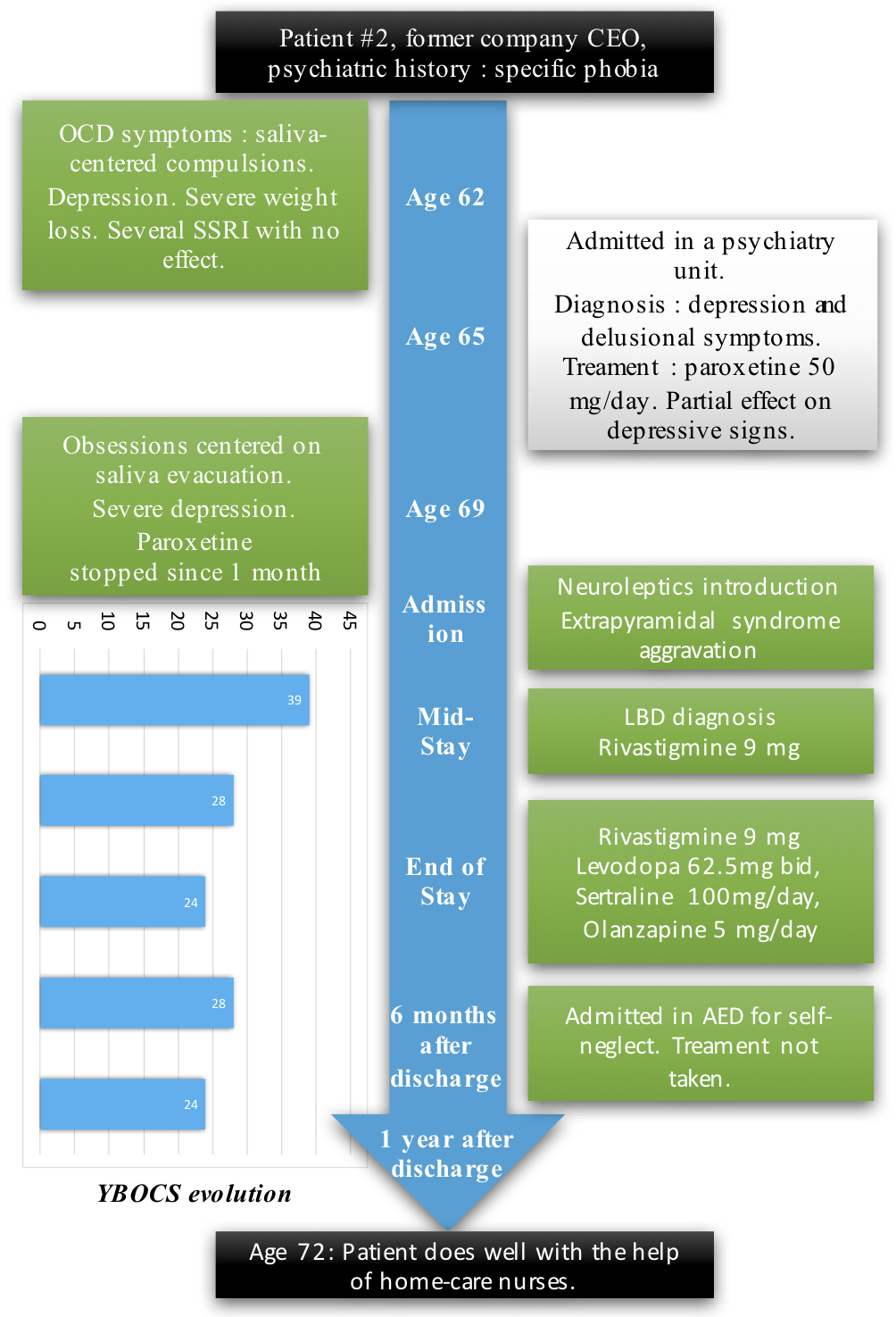

Supplement: Image 2 — Patient #2 Timeline: historical and current information from this episode of care. [file Image_2.jpeg]

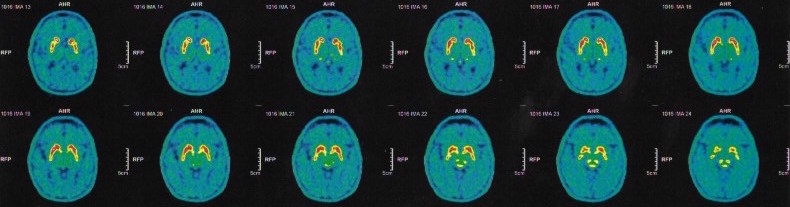

Supplement: Image 3 — Complementary sequences of DaT-SCAN SPECT from Patient #1, showing decreased dopamine concentrations in both putamen structures. [file Image_3.jpeg]

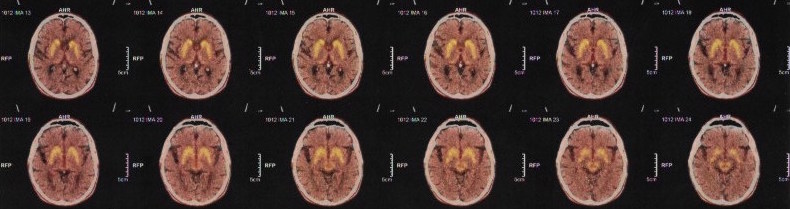

Supplement: Image 4 — Complementary sequences of DaT-SCAN SPECT from Patient #1, showing decreased dopamine concentrations in both putamen structures. [file Image_4.jpeg]

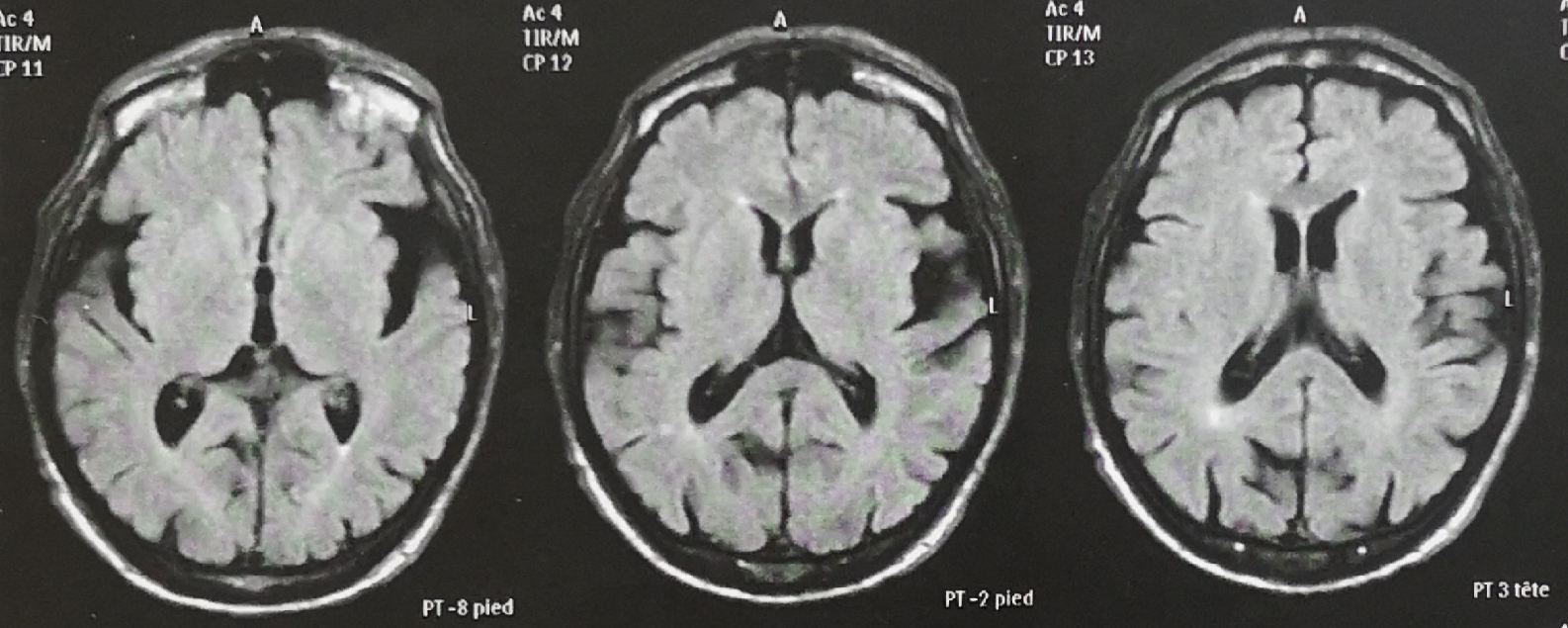

Supplement: Image 5 — Brain MRI from Patient #2 in 2014. Cerebral microbleed can be observed in left internal capsule. [file Image_5.jpeg]

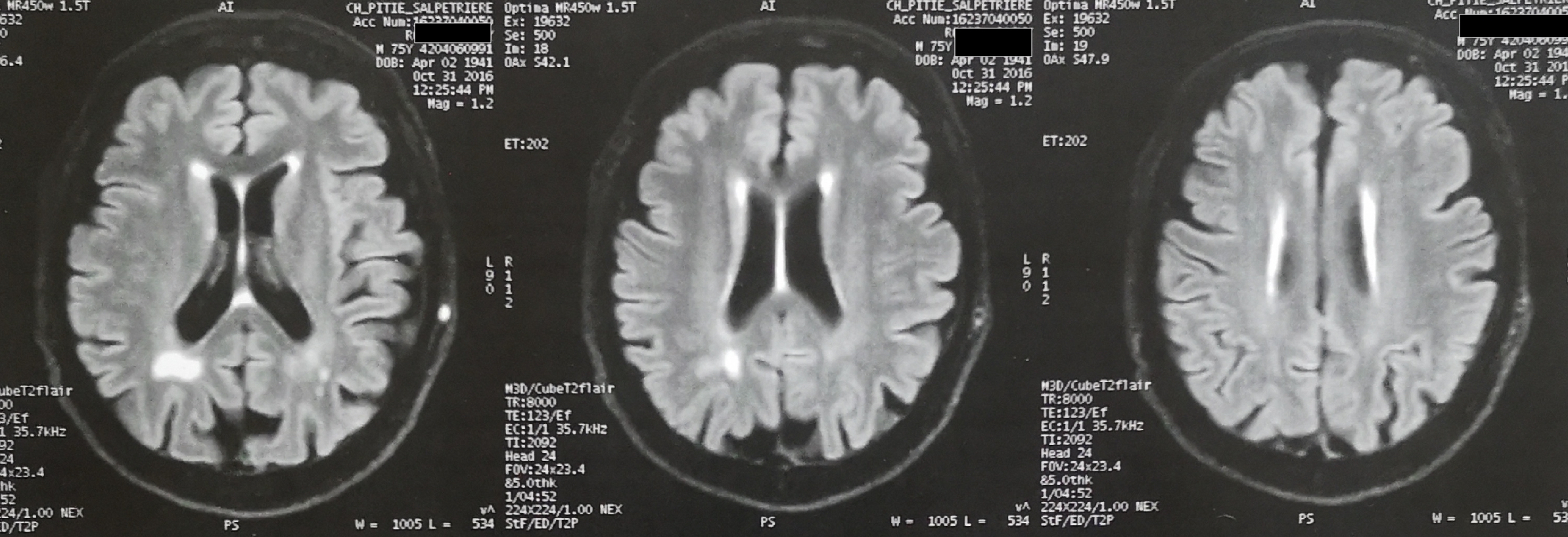

Supplement: Image 6 — Brain MRI from Patient #2 in 2016, at time of admission, showing cortical and sub-cortical atrophy. Superficial and deep cerebral microbleeds are observed. [file Image_6.jpeg]

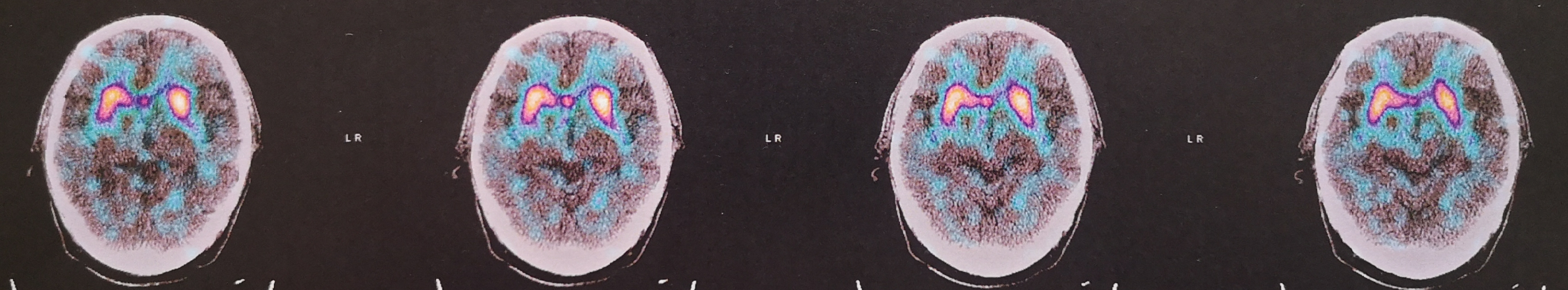

Supplement: Image 7 — Complementary sequences of DaT-SCAN SPECT from Patient #2, showing significant bilateral reduction in putamen uptake [file Image_7.jpeg]
