# Supplementary material for: Late-Onset OCD as a Potential Harbinger of Dementia With Lewy Bodies: A Report of Two Cases
Source: Front Psychiatry. 2020 Jun 30;11:554. doi: 10.3389/fpsyt.2020.00554 (PMC7344181; doi:10.3389/fpsyt.2020.00554)
Supplement: Table 1 — Patient#2 Neuropsychological Assessment in 2016. This assessment has been realized two months after patient's admission in psychiatry unit, once neuropsychiatric symptoms were stabilized (under rivastigmine 9 mg, olanzapine 5 mg and sertraline 100 mg daily). [file DataSheet_1.docx]

Imagery work-up patient #1

Brain MRI: A moderate leukopathy was observed on brain MRI (stage 2, Fazekas and Schmidt classification). It also showed a discrete cortico-cortical atrophy, predominantly posterior parietal. Hippocampus were grade 2 from Scheltens.

EEG: background activity was moderately slowed (7-8 Hz). It was symmetrical and moderately spatialized, reactive. Fast beta beta rhythms were recorded in the anterior regions.

PET-FDG found:

- a discrete bilateral prefrontal hypermetabolism in front of too good a visualization of the primary sensitivomotor areas.

- a frank bilateral hypometabolism of the parieto-temporo-occipital associative cortex, and of the primary visual cortex.

- a normal and symmetrical appearance of internal temporal structures and subcortical structures.

A symmetrical decrease in the tracer fixation was observed on the DaT-Scan, facing putamen, in favor of a pre-synaptic dopaminergic denervation (for complementary sequences, see Figure 3 and 4).

Imagery work-up patient #2

Brain MRI showed a cortico-subcortical atrophy without topographic predominance. Several superficial and deep micro bleeds could also be observed, evoking hypertensive micro angiopathy (2014 Brain MRI is shown in Figure 5 and 2016 Brain MRI is shown in figure 6).

Frank and bilateral decrease in striatal fixation of the predominant tracer in putamen was observed on the DaT-Scan, in favor of bilateral presynaptic dopaminergic denervation (see Figure 7 for complementary sequences).

Neuropsychological testing: what are the specific characteristics of each disease?

Deficits on tests of attention, executive functions and visuoperceptual ability occur early in DLB, while memory will be affected later in the typical course of the disease (40).

Patient #1 presented difficulty driving before consulting. He retired earlier than expected, while he had a brilliant career as university professor, which could be due to impaired job performance.

Mini-Mental State Examination (MMSE) is not reliable to differentiate among dementia types. However, the early appearance of impaired figure copying is suggestive of DLB (41). As early as 2014, Patient #2 presented anomalies in the copying of figures on MMSE test.

Early signs on neuropsychological testing also include deficits in tests of visuospatial and visuoperceptual abilities (42), that were observed in both Patients #1 and #2 (results listed below). Measures of executive functions and attention may also be impaired early on, which was observed for both patients as well(43).

Finally, when memory is impaired in DLB, memory retrieval may be more affected than acquisition (43). Patient #2 showed most difficulties in the recall sub-score of MMSE.

It is true that OCD itself is associated with neuropsychological deficits, some of which are common to DLB (44). Patients with OCD experience pronounced impairments in visuospatial memory (such as accurate recall of information about complex visual stimuli) and visual organizational skills (45,46). They also show deficits in executive functions, which have been considered core deficits in OCD, but that showed moderate effect in a meta-analysis on OCD cognitive profile (47). In this meta-analysis, the significant impairments to be observed were in planning ability, set shifting and response selection (such as inhibition of erroneous responses) (48,47). While planning ability showed medium-to-large effect size, set-shifting ability and cognitive inhibition showed medium or small-to-medium effect size (47).

Some features observed in Patients #1 and #2 that were typical for DLB cannot be caused by OCD *per se*. Patients with OCD have preserved attentional ability (47), while Patients #1 and #2 showed pronounced attentional impairments.

Finally, one could make the assumption that DLB, where cognitive dysfunction is often the presenting symptom, favored OCD development through executive and attentional disorders, enabling obsessions intrusion.

Neuropsychological testing scores from patient #1

*Unfortunately, some scores were not recorded in patient’s medical file.*

One month before admission (July 2016, treatment = paroxetine 20 mg / day), the following cognitive impairments were assessed in consultation.

Concentration and attention disorders were observed:

- the patient forgot to turn off the taps after washing rituals

- he lost his personal belongings in the different places he passed by

- he experienced troubles with planning and reasoning, being unable to carry out two tasks at the same time

- he showed a reduction in comprehension of complex sentences.

Executive functions disorders were observed at different levels :

- difficulty organizing his speech

- words findings difficulties.

There were also praxis disorders: ideomotor, constructive (in writing) and reflexive, with many simplifications and approximations.

These cognitive problems had probably settled insidiously over the last few years, as they were not the main complaint of the patient and his entourage.

On second neuropsychological testing (September 2016, before rivastigmine introduction), the following impairments were observed:

- impairment of storage processes in episodic memory (verbal anterograde memory)

- consolidation of long-term information was possible (no loss after delay).

Neuropsychological assessment found more characteristic impairments of DLB:

- attentional interferences with memory functioning (fluctuations in retrieval ability)

- a severe deficit of sustained attention, with frequent fluctuations and significant fatigability

- a dysexecutive syndrome, with working memory disorders, difficulties in motor programming, lexical evocation, conceptual abstraction, inhibition

- praxis disorders (ideomotor, visuo-constructive, visuo-spatial).

Two months later (November 2016), the patient showed the following cognitive profile:

- inaccurate spatial-temporal orientation

- MMSE = 24/30 (errors in words recall and in mental arithmetic)

- slowed literal fluency (12 words in 1 minute with perseverations)

- decreased abstraction capacity

- no visuo-spatial difficulties with entangled figures

- good color recognition

- dyscalculia

- no right/left indistinctness, no body schema disorder

- no praxis disorders.

In May 2017, the patient showed improved attentional abilities and marked alertness improvement during the day.

In June 2017, patient sees on a regular basis his speech therapist. During these consults, the following elements were observed:

- attentional disorder

- hypophonia with latency of verbal response

- verbal fluency: 12/12

- spatiotemporal orientation: 12/12

- mental manipulation abilities at 12/12

- naming abilities at 12/12

- visuoconstructive abilities at 12/12.

Most impairments were observed in learning skills (6/12), image recall (10/12) and problem solving (9/12).

In September 2017, patient’s cognitive profile is stable. Persistent attentional fluctuations are observed, as well as extrapyramidal syndrome and visual hallucinations. The patient remains autonomous for most activities of daily living.

In November 2017, the patient describes persistent attentional fluctuations, that improve after 6pm. He also relates confusion and sedation in the morning. He continues to experience attentional fluctuations in his bladder improvement, well-criticized visual hallucinations (objects or children perceived furtively), a moderate extrapyramidal syndrome, difficulties in reading the clock, severe orthostatic hypotension.

Neuropsychological work-up patient #2

Neuropsychological test carried out in May 2014, two years before admission in our unit, found a score of 24/30 on MMSE:

- orientation: 8/10

- registration: 3/3

- attention and concentration: 4/5

- recall: 1/3

- language: 8/8

- drawing: 0/1

Neuropsychological testing carried out in December 2016, while the patient was hospitalized and not yet treated for DLB, found :

- preservation of overall cognitive efficiency in MMSE (28/30)

- an efficient episodic verbal memory but with fragile consolidation.

- preserved temporo-spatial orientation.

More characteristics impairments of DLB were observed (49): weak executive functions, especially on the instrumental level. Visuospatial and visuo-perceptual capacities were altered. Complete work-up is provided in Table 1.

Thus, these difficulties pointed towards multifactorial cognitive disorders, with likely thymic participation, which did not explain the whole cognitive profile though.

Table 1. Patient #2 neuropsychological testing

*Times are provided in seconds. Percentiles provided are for same age and same education level.*

| **COMMON COGNITIVE TESTS** | |
| --- | --- |
| **MMSE** | 28/30 |
| Orientation | 9/10 |
| Registration | 3/3 |
| Attention and concentration | 5/5 |
| Recall | 2/3 |
| Language | 8/8 |
| Drawing | 1/1 (only on the second try) |
|  |  |

| **WORKING MEMORY** | | |
| --- | --- | --- |
| **WAIS IV** | Raw subject score | Norms in healthy population |
| Digit Span Forward | 7 | 7+/-2 |
| Digit Span Backward | 5 | 5+/-2 |
| Digit Span Sequencing | 5 | 5+/-2 |
|  | Scaled score | Norms for scaled score |
|  | 13 | 10 |
|  |  |  |

| **VERBAL EPISODIC MEMORY** | | |
| --- | --- | --- |
| **RL/RI-16** | Subject Score | Percentile |
| Identification | 16/16 |  |
| Encoding (immediate recall) | 15/16 | 25 |
| Free Recall 1 | 9/16 | 75 |
| Free Recall 2 | 7/16 | 25-50 |
| Free Recall 3 | 7/16 | 25 |
| Total Recall 1 | 15/16 | 50 |
| Total Recall 2 | 16/16 | 25 |
| Total Recall 3 | 14/16 | 5 |
| Free Total Recall | 23/48 | 50 |
| Free + Cued Total Recall | 45/48 | 50 |
| Reactivity to recall cues | 88% | 25-50 |
| Intrusions number | 5 |  |
| Free Delayed Recall | 10/16 | 50-75 |
| Total Delayed recall | 14/16 | 5 |
| Recognition | 16/16 |  |
| Wrong recognitions | 0 |  |
|  |  |  |
|  |  |  |
| **EXECUTIVE FONCTIONS** | | |
| **Trail-Making Test** |  |  |
| Trail A | Subject scores | Percentile |
| Time (seconds) | 81 | <25 |
| Errors | 0 | 95 |
| Trail B | stopped at 326 seconds  because of difficulty | |
| Time (seconds) | 326 | <5 |
| Errors | 3 (perseveratives) | <5 |
|  |  |  |
| **Verbal Fluencies** | Subject Score | Percentile |
| Phonemic (letter P, 2 minutes) | 22 | 50-75 |
| Semantic (animals, 2 minutes) | 20 | 10-25 |
|  |  |  |
| **Stroop Test** | Subject Results | Percentile |
| Colors naming time (sec) | 90 | 10 |
| Uncorrected errors in colors naming | 2 | <5 |
| Reading time (sec) | 47 | 50-75 |
| Uncorrected errors in words reading | 0 | 95 |
| Interference time (sec) | 218 | <5 |
| Uncorrected errors interference | 3 | <5 |
| Interference time-colors naming time (sec) | 128 | 10 |
| Uncorrected errors interference- uncorrected errors in colors naming | 1 | 10-25 |
|  |  |  |

| **SPEECH** | | |
| --- | --- | --- |
| **Oral language** | Subject Score | Norms in healthy population |
| **DO-80** | 74/80 | 76,2 |
|  |  |  |

| **GESTURAL PRAXIS** | | |
| --- | --- | --- |
| **Motor** | Subject Score |  |
| Bimanual coordination | 12/12 |  |
|  |  |  |
| **Ideomotor praxis (Mahieux)** | Subject Scores | Norms in healthy population |
| Symbolic gesture | 5/5 | >/= 4 |
| Action mimicry | 9/10 | >/= 8 |
| Abstract gesture | 4/8 | > 6 |
|  |  |  |

| **VISUOSPATIAL AND VISUOCONSTRUCTIVE  ABILITIES** | | |
| --- | --- | --- |
| **Rey Figure** | stopped at 99s | healthy score < 427 s |
|  | Subject Score | Norms in healthy population |
| **Cubes and triangles BEC96** | 10/12 | 10,73 |
|  |  |  |
| **Clock drawing, spontaneous** | Numbers misplaced in the circle | |
| **Clock drawing with copy** | Numbers misplaced in the circle | |
|  |  |  |
| **Visual Object and Space Perception battery (VOSP)** | Subject Scores | Cut-off Scores |
| Shapes detection | 19/20 | 15 |
| Points counting | 10/10 | 8 |
| Cubes analysis | 7/10 | 6 |
| Inachieved letters | 15/20 | 16 |
|  |  |  |

The patient hasn't had another neuropsychological testing since. Apart from attentional fluctuation, his cognitive profile remains stable according to the neurological follow-up.

References in text

**40.** Tiraboschi P, Salmon DP, Hansen LA, Hofstetter RC, Thal LJ, Corey-Bloom J. What best differentiates Lewy body from Alzheimer's disease in early-stage dementia? Brain J Neurol (2006) 129:729–35. doi: 10.1093/brain/awh725

**41**. Ala TA, Hughes LF, Kyrouac GA, Ghobrial MW, Elble RJ. Pentagon copying is more impaired in dementia with Lewy bodies than in Alzheimer's disease. J Neurol Neurosurg Psychiatry (2001) 70:483–8. doi: 10.1136/jnnp.70.4.483

**42.** Gurnani AS, Gavett BE. The Differential Effects of Alzheimer's Disease and Lewy Body Pathology on Cognitive Performance: a Meta-analysis. Neuropsychol Rev (2017) 27:1–17. doi: 10.1007/s11065-016-9334-0

**43.** Bradshaw JM, Saling M, Anderson V, Hopwood M, Brodtmann A. Higher cortical deficits influence attentional processing in dementia with Lewy bodies, relative to patients with dementia of the Alzheimer's type and controls. J Neurol Neurosurg Psychiatry (2006) 77:1129–35. doi: 10.1136/ jnnp.2006.090183

**44.** Oda H, Yamamoto Y, Maeda K. Neuropsychological profile of dementia with Lewy bodies. Psychogeriatrics (2009) 9:85–90. doi: 10.1111/j.1479- 8301.2009.00283.x

**45.** Okasha A, Rafaat M, Mahallawy N, El Nahas G, El Dawla AS, Sayed M, et al. Cognitive dysfunction in obsessive-compulsive disorder. Acta Psychiatr Scand (2000) 101:281–5. doi: 10.1034/j.1600-0447.2000.101004281.x

**46.** Choi J-S, Kim SH, Yoo SY, Kang D-H, Kim C-W, Lee J-M, et al. Shape deformity of the corpus striatum in obsessive-compulsive disorder. Psychiatry Res (2007) 155:257–64. doi: 10.1016/j.pscychresns.2007.02.004

**47**. Shin NY, Lee TY, Kim E, Kwon JS. Cognitive functioning in obsessivecompulsive disorder: a meta-analysis. Psychol Med (2014) 44:1121–30. doi: 10.1017/S0033291713001803

**48.** Chamberlain SR, Blackwell AD, Fineberg NA, Robbins TW, Sahakian BJ. The neuropsychology of obsessive compulsive disorder: the importance of failures in cognitive and behavioural inhibition as candidate endophenotypic markers. Neuro s c i Biobehav Rev (2005) 29:399–419 . d o i : 10.1016/ j.neubiorev.2004.11.006

**49.** Mak E, Su L, Williams GB, O'Brien JT. Neuroimaging characteristics of dementia with Lewy bodies. Alzheimers Res Ther (2014) 6:18. doi: 10.1186/ alzrt248

French neuropsychological tests references

**RL-RI 16**

Amieva, H., Carcaillon, L., L'Alzit-Schuermans, P. R., Millet, X., Dartigues, J. F., & Fabrigoule, C. (2007). *Test de rappel libre/rappel indicé à 16 items: Normes en population générale chez des sujets âgés issues de l'étude des 3 Cités*. Cued and uncued memory tests: Norms in elderly adults from the 3C epidemiological study. Revue Neurologique, 163(2), 205-221. doi: 10.1016/S0035-3787 (07)90392-6

**DO-80**

Deloche, G. et Hannequin, D., DO 80: *Test de dénomination orale d’images*, Paris éditeur, Les Éditions du Centre de Psychologie Appliquée, 1997.

**Praxis Test, Mahieux**

*Validation d’une batterie brève d’évaluation des praxies gestuelles pour consultation Mémoire. Évaluation chez 419 témoins, 127 patients atteints de troubles cognitifs légers et 320 patients atteints d’une démence.* Validation of a brief screening scale evaluating praxis abilities for use in memory clinics. Evaluation in 419 controls, 127 mild cognitive impairment and 320 demented patients*.* F. Mahieux-Laurent et al*.* Revue Neurologique, Volume 165, n° 6-7, pages 560-567 (juin 2009), doi : 10.1016/j.neurol.2008.11.016

**BEC96**

*Batterie d’évaluation cognitive, Evaluation des troubles de mémoire et des désordres cognitifs associés*

Jean-Louis Signoret et al, <http://fmc31200.free.fr/MG-liens/Neurologie/bec96.pdf>

**PEGV**

*Protocole d’évaluation des gnosies visuelles.* Agniel et al. 1992
